# Video Game-Based Trunk Exercises for Rehabilitation in Chronic Stroke Survivors: A Mixed-Methods Feasibility Study

**DOI:** 10.3390/s24216830

**Published:** 2024-10-24

**Authors:** Norah A. Alhwoaimel, Ann-Marie Hughes, Martin Warner, Aqeel M. Alenazi, Mohammed M. Alshehri, Bader A. Alqahtani, Ahmed S. Alhowimel, Richard Wagland, Simon Brown, Ruth Turk

**Affiliations:** 1 Department of Health and Rehabilitation Sciences, Prince Sattam Bin Abdulaziz University, Al-Kharj 11942, Saudi Arabia; 2 School of Health Sciences, University of Southampton, Southampton SO17 1BJ, UK; 3 Department of Physical Therapy, Jazan University, Jazan 45142, Saudi Arabia

**Keywords:** stroke rehabilitation, video games, game-based rehabilitation, virtual reality, trunk exercise, trunk control, trunk impairment

## Abstract

**Aim:** To assess the feasibility of video game-based trunk exercises using the Valedo^®^ system in a chronic stroke population. **Method:** Ten chronic stroke survivors (eight males and two females, mean age 63 ± 15 years) were asked to complete 18 intervention sessions, each lasting 45 min., over 6–8 weeks. Feasibility was evaluated quantitatively using the Psychosocial Impact of Assistive Devices Scale (PIADS) as well as through recruitment, retention, adherence, and safety measures. Qualitative data on feasibility were collected through post-intervention semi-structured interviews. Descriptive analysis was used to summarize participant characteristics, recruitment, retention, and adherence. Qualitative data were analyzed using thematic analysis of the interviews. **Results:** Twelve stroke survivors were recruited from Southampton (United Kingdom) and Riyadh (Kingdom of Saudi Arabia), with two participants dropping out after the baseline assessment session. The remaining ten participants completed the study with a mean adherence of 96.11% to the planned sessions. No serious adverse effects were reported, however, four participants did experience trunk muscle tightness and fatigue. Post-intervention interviews revealed that participants encountered some physical and cognitive challenges while playing the Valedo video games. However, they felt that the implementation of trunk exercises using video games was safe, as the exercises were performed in a secure environment and in safe positions. **Conclusions:** The findings suggest that the Valedo system is feasible for delivering trunk exercises to chronic stroke survivors. Several factors should be considered when implementing this type of intervention in the future.

## 1. Introduction

Trunk control is an important predictor of outcomes of balance, gait, and activities of daily living (ADL) post-stroke [1,2,3]. The percentage of the variance of functional recovery after a stroke is explained by trunk control, ranging from 45% to 71% [2,3]. Trunk performance is commonly affected post-stroke, leading to impairments such as muscle weakness, loss of coordinated muscle action, overactive muscles, and stiffness [4,5,6]. These impairments can interfere with the ability to carry out ADL [3].

A systematic review and meta-analysis of 467 randomized control trials (RCTs) suggested that most rehabilitation programs after a stroke focus on upper limb (UL) or lower limb (LL) function [7]. However, there has been increasing research on trunk rehabilitation over the last decade [8,9,10,11,12,13,14]. The findings from these systematic reviews suggest that trunk exercise, either alone or as an additional therapy to conventional rehabilitation, can improve post-stroke trunk impairment at acute, subacute, and chronic stages.

The trunk exercises described in previous studies are repetitive standard exercises without targets and feedback (i.e., standardized objective goals and methods of assessing movements and conveying these to both the participant and the therapist). While repetitive exercises are essential for relearning and improving movements post-stroke, their repetitive nature can decrease participant motivation, affecting adherence to the rehabilitation program [15]. A systematic review of 31 qualitative studies found that stroke survivors reported negative experiences, such as disempowerment, boredom, and frustration, during physical rehabilitation [16]. Participants associated irrelevant tasks with boring or meaningless therapy and desired enjoyment from therapy through activities like Wii computer games or circuit classes. Using technology and virtual reality (VR) game-based rehabilitation may help create an enjoyable environment in which patients can perform motor skills, keeping them motivated and engaged in exercises [17,18].

Implementing VR in rehabilitation can involve either immersive VR, which uses a 3D environment to give patients a strong sense of presence, or non-immersive VR, where patients interact with visual and auditory stimuli displayed on a screen, such as video games [19]. Using VR as an intervention tool in stroke rehabilitation offers several benefits. First, VR enables patients to practice the tasks in a safe, controlled environment tailored to their needs, which gives the therapist control over the environment compared to real-life environments [20]. VR games also provide audio, visual, and haptic feedback, which is critical for improving motor learning and task performance [21]. Another key advantage is the ability to customize the difficulty of games, maintaining patients’ engagement by introducing challenges while considering their abilities [22].

A randomized controlled trial was conducted on chronic stroke patients with poor sitting balance investigated the effect of an intensive trunk control training program (10 sessions per week for 4 weeks) using video games on trunk control. The results post-training showed significant improvements in the Trunk Impairment Scale (TIS) and its subscales (*p* < 0.05) [23]. Another study verified these findings when comparing the effect of conventional exercise with driving-based interactive video games (DBIVGs) on trunk control in people with chronic stroke. Their results also demonstrated a significant improvement in the TIS and coordination subscale when comparing between groups (*p* < 0.05) [24]. However, the limitations of the suggested interventions include the use of an expensive device that requires large space for installation, thereby incurring high healthcare costs and making it difficult to apply in clinical settings [23]. Additionally, the video games used in previous studies [24,25] require a high level of UL motor function to be able to handle the device. Commercial VR devices (such as Nintendo Wii and Microsoft Kinect) have been suggested as alternatives for use in clinical settings [26,27,28]. However, these commercial VR devices also have limitations, including the requirement for specific UL dexterity to handle a Wii controller, a high initial level of balance to stand on the Wii balance board, and the non-adjustability of the difficulty level of the games [27,28,29].

In clinical settings, there is a need for commercial technologies that can be tailored to different disability levels, capture small compensatory movements during exercise, require only a small installation space, and are affordable. Therefore, this study aims to address these limitations by investigating the feasibility of video game-based trunk exercises using Valedo^®^ system in chronic stroke populations. The Valedo sensor system was originally developed as an assessment tool to measure trunk movements in healthy adults and as an intervention tool to treat low back pain through video game exercises [30,31]. This technology has the potential to be used as a tool for performing trunk exercises but requires an evaluation of its feasibility for people with chronic stroke to inform a large-scale RCT.

## 2. Methods

This study was a single-group, mixed-methods convergent parallel design approved by the University of Southampton Ethics Committee (Ethics number: 30748). A sample of convenience was recruited from Southampton (United Kingdom) and Riyadh (Kingdom of Saudi Arabia). Several methods of recruitment, including email invitations, advertising posters, and visits to clinical centers (Prince Sultan Humanity City, Al-Faran Medical Center, and MYO Osteopathy Center), were used. 

### 2.1. Participants

Individuals with chronic stroke and mild to severe trunk impairment who were not currently undergoing any form of therapy were eligible to participate in the study. Individuals were excluded if any of the following applied to them: (1) currently receiving neurorehabilitation, (2) total hip replacement (THR), (3) cognitive impairment, and/or (4) unable to commit to the 18 sessions of the trunk exercise program. THR was excluded due to the contraindicated movements involved in the training, such as twisting and crossing the legs, while cognitive impairment was excluded because the ability to understand instructions is necessary for interacting with video games [32].

### 2.2. Study Setting and Procedure

**Figure 1:**
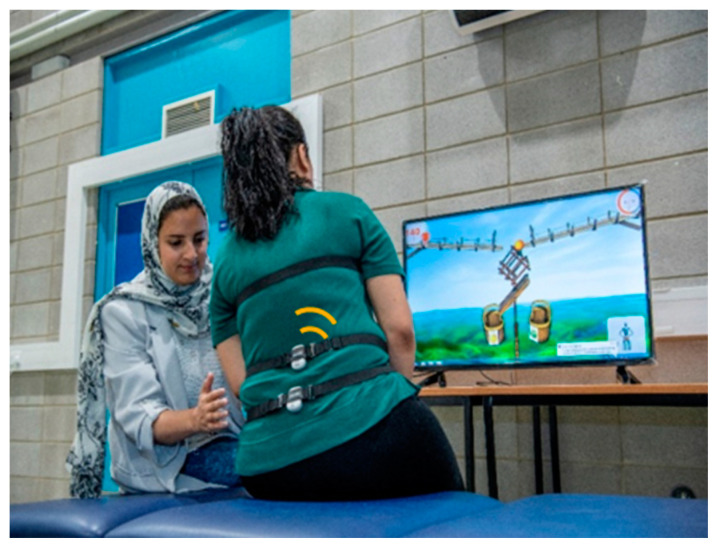
Study set-up: participant sitting on the bed and playing Valedo^®^ games.

The Valedo^®^ system (Hocoma AG, Volketswil, Switzerland) is a wireless movement analysis tool that uses three lightweight sensors equipped with inertial measurement units (IMUs). These sensors measure trunk movement (in degrees) and the velocity of body segments relative to magnetic fields and gravity in a non-invasive way. The system includes three lightweight sensors, a Bluetooth dongle, a USB stick with software, a charging cable, and a computer with at least 4 GB of space. The sensors are attached to the trunk using three adjustable belts (Figure 1).

It was originally developed for treating low back pain through virtual reality (VR) technology to enhance body awareness of the lower back [30,31]. It has been validated as an assessment tool for measuring trunk movement in both chronic stroke patients and age-matched healthy participants, demonstrating acceptable levels of validity and reliability [33,34]. Additionally, the Valedo^®^ system can be used as an intervention tool. It features a software package with video games designed for trunk training, which can be tailored to each participant’s trunk range of motion (ROM). This includes trunk forward flexion/extension, trunk lateral flexion, trunk rotation, pelvic lateral tilt, and pelvic sagittal tilt. A description of each game is presented in Supplementary Materials Table S1.

Each participant received a total of 18 sessions, divided into three sessions per week (45 min/session) within a period of 6–8 weeks. The participants could practice the exercise either from sitting or standing position.

The video game program was personalized for each participant according to their ability based on their Trunk Impairment Scale (TIS) and Berg Balance Scale (BBS) results. The TIS and BBS scores were obtained from each participant at baseline by following the standardized measurement procedures for each tool before designing the intervention program [6,35]. Participants with BBS ≥45 practiced trunk exercise from a standing position, while those with lower BBS practiced the exercise from a sitting position. In addition, TIS was used to determine the content of the trunk exercise program. For example, participants who could stand alone safely with a BBS score of ≥45 and had a full static sitting subscale but experienced decreased dynamic balance in TIS due to limited pelvic shortening/lengthening ability practiced the exercises from both sitting and standing positions. The goal of the sitting exercises was to improve the dynamic subscale by practicing pelvic tilting exercises while playing golf and glider games (see Supplementary Materials Table S1). A detailed description of the games prescribed for each level according to TIS and BBS results is presented in Supplementary Materials Table S2.

Exercise progression involved changing the training position and increasing game difficulty based on the scores achieved at the end of each game and changes in the BBS score. Each Valedo game has three difficulty levels: easy, medium, and hard. Participants started at the easy level and progressed to the next level upon achieving the maximum score for that level. Additionally, participants’ balance was reassessed using the BBS at the mid-training period (ninth session). Progression from sitting to standing exercises was based on two criteria:

1. Participants achieving a high balance score (BBS > 45) during the mid-treatment reassessment (ninth session);
2. Participants achieving a full score in three games (one targeting the static sitting balance subscale, one targeting the dynamic sitting balance subscale, and one targeting the coordination subscale), even before the mid-treatment period.

### 2.3. Outcome Measures

Feasibility studies can address eight different focus areas: acceptability, demand, implementation, practicality, adaptation, integration, expansion, and limited-efficacy testing [36]. For the purpose of this study, the acceptability and implementation of delivering trunk exercise using video games in people with chronic stroke were tested [36]. Acceptability can be defined as “A multi-faceted construct that reflects the extent to which people delivering or receiving a healthcare intervention consider it to be appropriate, based on anticipated or experienced cognitive and emotional responses to the intervention” [37]. Additionally, implementation focuses on the extent and manner in which an intervention can be implemented as planned and proposed in an uncontrolled study design [36]. Both acceptability and implementation can be measured either quantitatively (e.g., satisfaction scale and adherence level) or qualitatively through participant interviews.

For the acceptability domain, the Psychosocial Impact of Assistive Devices Scale (PIADS) was used post-intervention. PIADS is self-rating questionnaire comprising 26 items, measures the psychological impact by addressing the following three indicators: competence, adaptability, and self-esteem [38]. Scores can range from −3 (maximum negative impact) through zero (no perceived impact) to +3 (maximum positive impact). It has well-established psychometric properties [39] and clinical utility [40] and has been shown to be a sensitive and responsive measurement tool to assess the impact of several assistive devices across various disability populations [41,42].

For the implementation domain, recruitment and retention rates, adherence to the intervention, and adverse events were measured [43]. Recruitment was defined as the ratio of invited stroke patients who agreed to participate in the study to those who did not. Retention was determined by the proportion of patients who completed the trunk rehabilitation program [44]. Additionally, adherence was considered to be the compliance of participants in attending sessions, regardless of their behavior during the sessions. It was measured using an adherence index, calculated by dividing the number of sessions attended by the number of planned sessions [45]. Lastly, safety was monitored in every session by recording any events that occurred and by asking participants at the beginning of each session if they experienced any falls, pain, or other symptoms since the last exercise session. Furthermore, this study investigated the feasibility of the Valedo games to deliver trunk exercises through qualitative data obtained from semi-structured interviews conducted post-intervention. The semi-structured interview guide comprised eight categories of questions, including attractiveness and enjoyment of the program, impact of intervention, motivation to exercise, influence of audio–visual feedback, safety, commitment to the program, usability of the system, and general questions for improving the study. The questions were designed as open-ended with prompting questions, and Likert scale questions were used to assess the difficulty of each game played. Each participant was interviewed by the researcher after completing the intervention.

### 2.4. Data Analysis

Descriptive analysis was used to summarize the participants’ baseline characteristics (age, sex, stroke onset, hemiplegic side, TIS, BBS, and assistive device use). PIADS scores were not normally distributed, and the sample size was small; therefore, a non-parametric test (median) was used.

Semi-structured interviews were transcribed verbatim by the lead researcher and analyzed using the framework approach [46]. The NVivo12 qualitative data software package (QSR International Pty Ltd., Chadstone, VIC, Australia) was used to manage the qualitative data and facilitate the process of coding transcripts.

## 3. Results

### 3.1. Participants Characteristics

The study’s sample included eight males and two females, with a mean age of 63 ± 15 years. Five participants were recruited from Southampton, UK, and five participants were recruited from Riyadh, KSA. The baseline clinical outcome measures presented in Table 1.

### 3.2. Quantitative Results

The quantitative results for acceptability and implementation were assessed using PIADS, study retention rates, safety, and adherence to the intervention. 

#### Psychological Impact

**Figure 2:**
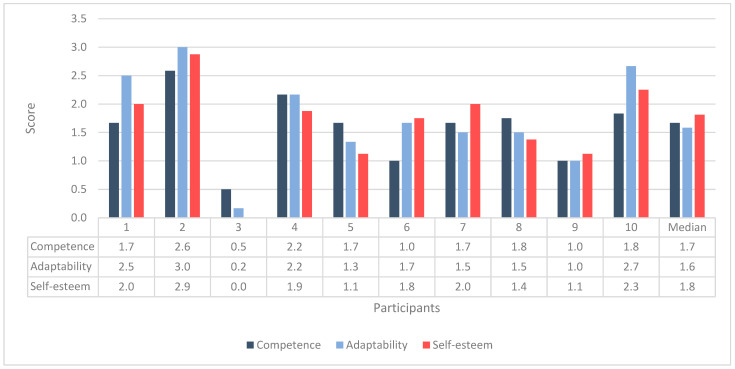
Bar chart displaying PIADS ratings for each participant.

The PIADS was measured for each participant at the end of the intervention program, as illustrated in Figure 2. The ratings reflect the three subscales of the PIADS: competence, adaptability, and self-esteem. The median scores for the PIADS subsections—competence (median = 1.7), adaptability (median = 1.6), and self-esteem (median = 1.8)—indicated that participants generally experienced positive psychosocial outcomes (maximum positive impact = 3) from using video games for trunk exercises.

However, P3 had the lowest total score (total PIADS = 7), with most of his responses indicating no perceived impact. The only item reported as negative (maximum negative impact = −3) by three participants (P1, P4, and P8) was “frustration”, as shown in Table 2. Scores of −2 and −3 in frustration indicate that these participants were upset about their lack of progress or felt disappointed.

#### Recruitment and Retention

The recruitment process took place between April 2018 and April 2019 in Southampton (UK) and between January and May 2019 in Riyadh (KSA). In the UK, six chronic stroke participants were enrolled in the study (five recruited from the Health Sciences School registry and one recruited from a stroke club in Southampton). In Saudi Arabia, six chronic stroke patients were enrolled in the study (three recruited from Al Faran Medical Center and three recruited from Prince Sultan Humanity City).

**Figure 3:**
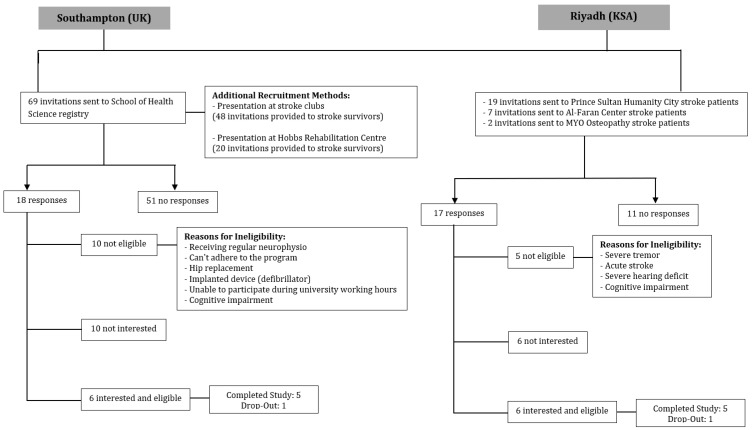
Recruitment flowchart.

A total of 27 patients expressed interest in participating in the study, but only 12 of them (44.44% of the interested participants) were eligible to participate in the study, as presented in the recruitment flowchart (Figure 3). The reasons for exclusion included the inability to adhere to the program due to logistical reasons (i.e., transportation, distance, and frequent travel), regular neurophysio treatment, hip replacement, acute stroke, severe hearing deficit, cognitive impairment, severe tremors and balancing problems, an implanted device (defibrillator), and the inability to attend the whole program due to the busyness of the caregiver during working hours. Two participants (17% of the enrolled participants) dropped out after the initial assessment (one in Southampton and one in Riyadh), while ten participants (83% of the enrolled participants) completed the study. The reasons for dropout were the content of the intervention program (participant thought that the program included lower limb exercise) and travel.

#### Adherence

The number of sessions attended by each participant was recorded and is detailed in Table 3. Each participant received a table for all scheduled appointments in the first session and an amendment on the date and time applied according to the participant’s convenience. All participants completed the pre- and post-assessments, but only eight out of ten participants attended all planned sessions within six to eight weeks. The remaining two participants (P3 and P9) were each only able to complete 17/18 and 12/18 sessions due to sickness (P3) and a busy caregiver (P9). In general, the percentage of attendance was 96.11%, as presented in Table 1. All participants completed the planned time for each exercise session (45 min of actual training).

#### Safety

No major adverse events were reported. Two participants (P3 and P10) reported fatigue at the end of the day after the trunk exercise. One participant (P3) mentioned that he experienced mental fatigue more than physical fatigue. Three participants (P4, P8 and P10) reported that they felt tightness in the lateral trunk muscle of the affected side at the end of the day when playing the Clock game (the game required lateral trunk flexion on both sides) in the initial few sessions. The tightness disappeared in the last two weeks of intervention and did not affect their sleep or daily activities.

### 3.3. Qualitative Results

**Figure 4:**
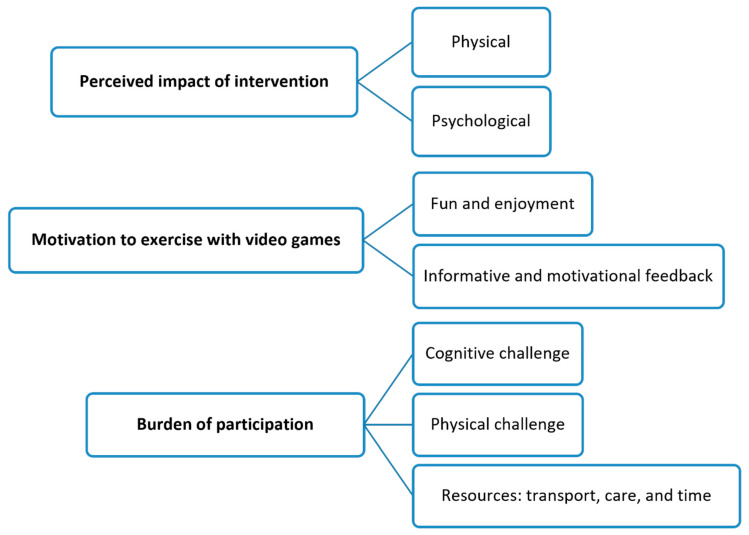
Thematic map of the acceptability of delivering trunk exercises using the Valedo video game system.

**Figure 5:**
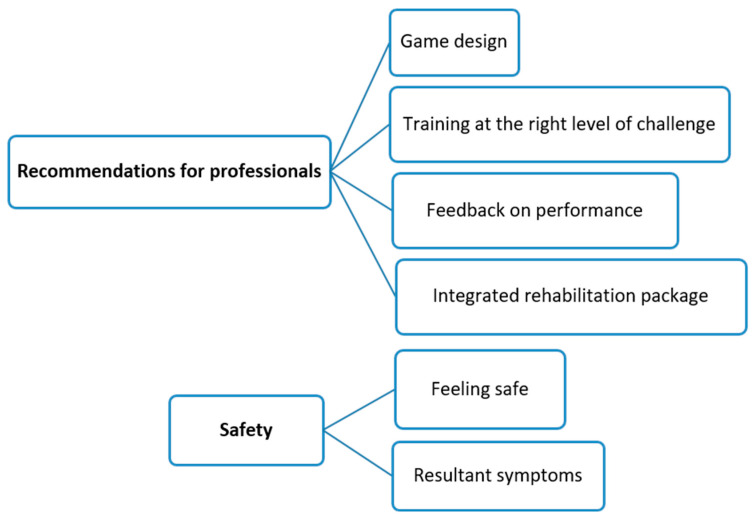
Thematic map of implementation of trunk exercises using the Valedo video game system.

Following the framework analysis approach, all raw data were indexed to the appropriate theme to explore feasibility. The themes are presented in two thematic maps in Figure 4 and Figure 5. The feedback from participants supported the quantitative findings by explaining in depth the factors affecting the feasibility of the study protocol and intervention. Participants were identified by their participant ID and age, as shown in Table 1.

#### Acceptability of Trunk Exercises Using the Valedo Video Game System

Throughout the interviews, participants shared their experiences with using video games for trunk exercises and discussed the acceptability of this rehabilitation method. Three key themes emerged regarding the use of Valedo video games as a tool for trunk exercise therapy: (1) perceived impact, (2) motivation to exercise with video games, and (3) burden of participation.

1. **Perceived impact**

**Physical impact**

By the end of the intervention, participants reported noticeable improvements in daily activities and mobility. One participant stated, “*My leg felt lighter when I walked*” (P2, 54), while another shared, “*My daily activities have changed 70% for the better… I can do that by myself*” (P7, 75). Improved balance was also highlighted: “*I’ve been able to move around in my boats a little bit easier*” (P1, 49). Posture and confidence improved, with one participant noting, “*I walk with a straight back… I’m more confident and better balanced*” (P8, 40). Additionally, participants experienced reduced muscle tightness. As one participant stated, “*The muscle tightness in my sides has significantly decreased*” (P10, 54).

**Psychological impact**

The psychological impact of using Valedo video games for trunk exercises was significant, with participants reporting reduced fear of falling, increased confidence, and improved cognitive function. One participant shared, “*At first, I felt scared of falling, but over time the feeling went away*” (P10, 54). Others noted feeling stronger and more capable: “*I’m more confident in my movements*” (P5, 59). The sense of hope and excitement was also highlighted, with one participant stating, “*It opened my eyes to more possibilities*” (P1, 49). Improved focus and multitasking abilities further enhanced engagement: “*It helped me mentally in terms of focus*” (P7, 75).

2. **Motivation to Exercise with Video Games**

Participants were motivated to exercise with Valedo video games due to the fun and enjoyment as well as the informative feedback given by the system.

**Fun and Enjoyment**

Most participants enjoyed using the Valedo system. One participant said, “*I enjoyed it immensely*” (P4, 69), while another shared, “*It felt like I was having fun and exercising at the same time*” (P10, 54). There was an agreement among participants that they would not engage in the exercises if it involved repetitive movements without the video games, as traditional exercises were perceived as boring. As one of the participants said, “*You just wouldn’t do those exercises without the games. It would be nothing but repetitive boredom, and I know I wouldn’t stick with it*” (P3, 62).

**Informative and Motivational Feedback**

The real-time feedback, such as visual and auditory cues, motivated participants. One shared, “*The audio and visual feedback gave me a sense of success*” (P8, 40), while another said, “*I loved seeing my movements reflected on the screen; it reassured me that my body was indeed moving*” (P7, 75). This feedback helped participants to set goals and stay motivated throughout the sessions.

3. **Burden of Participation**

The burden of participation in the Valedo trunk exercise program emerged as a significant theme, with participants facing cognitive, physical, and logistical challenges.

**Cognitive Challenge**

Multitasking games required high concentration and mental effort. As one participant described the Break Breaker game, “*That was a lot of problem-solving and predicting where the ball is going to be*” (P1, 49). The unpredictability of the Fruits game made it cognitively challenging, requiring participants to maintain high concentration and react quickly to catch the fruit. One participant shared, “*It needs a great deal of mental effort. I couldn’t anticipate which fruit would come, whether orange or watermelon. So, I had to focus and move quickly towards the goal. Otherwise, I would lose it*” (P9, 75).

**Physical Challenge**

Several participants found the physical aspects of the exercises, such as trunk rotation and pelvic tilting, difficult. The standing position with the arms on the sides was felt to be physically challenging for participants with complete arm paralysis, especially when practicing trunk rotation exercise (i.e., Break Breaker game). One noted, ‘*I found that quite hard at first because I had my hands down by my side, which I thought my right arm was getting in the way. So, when I started doing it with my arms across my chest, I found it much easier*’. (P2, 54). Additionally, lower trunk exercises, like pelvic tilting, were considered difficult and unfamiliar by participants. One participant noted, “*It is hard to move my pelvis backwards and forwards. I didn’t normally practice this pelvic movement in my daily life*” (P8, 40).

**Resources: Transport, Care, and Time**

Logistical factors, including transportation and time management, were also challenging. As one participant stated, “*My sons drove me to the clinic, but sometimes they were too busy*” (P9, 75). Caregivers played a key role in helping participants commit to the intervention program. One participant expressed gratitude, saying, “*It was easy for me to commit to the sessions… thank God for my wonderful sons. If one couldn’t drive me to the clinic, the other would step in*” (P7, 75).

#### Implementation of Trunk Exercise Using Valedo Video Game System

Participants discussed factors that helped or hindered their practice of trunk exercises using the Valedo video game system. Two main themes emerged: (1) recommendations for professionals and (2) safety.

1. **Recommendations for Professionals**

Participants provided feedback for physiotherapists and game developers to improve the implementation of video game-based trunk exercises. This included subthemes of game design, level of challenge, performance feedback, and integrated rehabilitation.

**Game Design**

The design of certain games, like the Fruits game, was challenging for some participants due to unclear movements between the upper and lower trunk. One participant shared, “*‘Well, it’s as soon as I made the distinction that the top part was my trunk… I got my head around that, then I could suddenly decide which way to go and how far to lean’* (P1, 49). Additionally, the randomness of the Fruits game was insufficient, leading to repetitive movements on one side. Another participant suggested, “*I would introduce more randomness in the Fruit game*” (P4, 69).

The Colors game was difficult for all participants, as it required precise trunk movements. One noted, “*It’s so unforgiving. If you overextend or if you move too quickly, it’s really difficult to control’* (P5, 59). Furthermore, games like the Clock game were perceived as monotonous and repetitive, with one participant calling it “*boring because it was just going backwards and forwards*” (P3, 62).

**Training at the Right Level of Challenge**

Participants found that the challenge level was important, but some games needed to consider other factors like muscle flexibility. Two participants found the Clock game difficult due to muscle tightness, with one saying, “*It was difficult because I felt tightness in my muscles*” (P10, 54). Additionally, the Break Breaker game was too fast for some participants, who struggled to keep up with the rapid changes in the ball’s movement direction. One participant noted, “*Following the ball movement was exhausting, and it required very fast reactions*” (P9, 75).

**Feedback on Performance**

Participants found performance feedback helpful for setting goals. However, some struggled to understand the scoring system. One participant shared, “*I didn’t necessarily understand where the scores came from*” (P3, 62). Others understood it better after explanations from the physiotherapist. Additionally, the scoring system in the diver game frustrated one participant, as a small mistake would result in losing a significant number of points, making it his least favorite game. He explained, “*If I made a small mistake, I lost everything I had earned. This was really upsetting*” (P8, 40).

**Integrated Rehabilitation Package**

Two participants expressed a desire for the rehabilitation program to extend beyond the 18 sessions, with one stating, “*I wished the sessions were longer because I loved them*” (P10, 54). Additionally, some participants suggested a more comprehensive approach, incorporating exercises for both the upper and lower limbs alongside the trunk exercises. As one participant explained, “*I recommend developing a comprehensive program that combines video game-based trunk exercises with treadmill walking and therapeutic exercises for a hemiplegic hand and leg*” (P8, 40).

2. **Safety**

Safety was another key factor in implementing the Valedo video game system. Participants generally felt safe during exercise, with minimal symptoms reported.

**Feeling Safe**

Most participants felt safe due to the controlled environment and safe exercise positions. One participant stated, “*I feel perfectly safe while playing the video games. I know there’s nowhere to fall*” (P4, 69). Additionally, knowing that a physiotherapist was nearby increased participants’ sense of security. One participant shared, “*I knew the physiotherapist was there, so I felt much safer*” (P8, 40).

**Resultant Symptoms**

Two participants reported symptoms from specific exercises. One felt muscle tightness during the Clock game, stating, “*The tightness I felt on the sides made this exercise difficult*” (P8, 40). Another participant experienced dizziness during the Break Breaker game, commenting, “*Following the ball movement made me feel dizzy, so I needed to pause*” (P9, 75).

## 4. Discussion

The objective of this study was to explore the feasibility of using the Valedo video game-based system to deliver trunk exercises for people with chronic stroke. The findings indicated that the Valedo system was acceptable to this population and could be implemented in clinical settings.

### 4.1. Acceptability of the Valedo System

Participants found the Valedo video games an acceptable method for delivering trunk exercises. Our PIADS results demonstrated a significant positive impact, with participants experiencing high positive psychosocial outcomes from using video games for trunk exercises across all PIADS subsections: competence, adaptability, and self-esteem. Compared to a previous study that used technology for upper limb rehabilitation post-stroke, our results were higher. The previous study reported a moderate positive impact of computer games for upper limb rehabilitation on measures of competence (+1.01), adaptability (+1.1), and self-esteem (+0.46) [47].

Acceptability was linked to the enjoyment experienced while exercising with the Valedo system, which addressed the boredom associated with traditional exercises. Participants widely reported that conventional repetitive trunk exercises were boring, and they would not engage in them without the video games. The use of video games mitigated this boredom, with some participants becoming so engaged that they lost track of time. This aligns with the concept of “flow”, a state of intense focus and enjoyment during an activity [48]. This finding is consistent with previous studies using Microsoft Kinect video games for balance rehabilitation post-stroke, where participants also found video game-based rehabilitation enjoyable [49,50].

Although this study was not designed to measure efficacy, the qualitative results showed positive changes in physical and psychological aspects, which motivated participants to continue exercising with the Valedo system. These findings could be interpreted in light of the self-determination theory (SDT) [51]. According to SDT, the adoption and maintenance of a behavior is influenced by valuing or enjoying the behaviors [51]. Thus, the enjoyment reported while exercising using the Valedo system and the perceived physical and psychological impact indicate the possibility of video game-based trunk rehabilitation in improving exercise adherence.

Adherence to therapeutic interventions is often linked to acceptability [52]. In this study, adherence was high, with 80% of participants completing the program. Health status and the availability of carers were primary reasons for not attending some sessions. This is in agreement with findings by Proffitt and Lange, who identified that insufficient time and family support can be a barrier to adhering to a physical therapy program [50]. Furthermore, previous literature has identified changes in health status as a major barrier to adherence [53,54].

The recruitment process for the current study was somewhat slow; however, the eligibility rate was high compared to previous studies, where over 40% of screened participants were excluded due to motor ability limitations [55,56]. The high eligibility rate in our study can be attributed to the Valedo system’s suitability for various levels of disability. Unlike other systems, the Valedo system does not require specific UL abilities to hold game controllers or a high level of dynamic balance to perform the VR exercises.

### 4.2. Implementation of the Valedo Video Games in Rehabilitation

The Valedo system was feasible even for participants with moderate disabilities, including wheelchair users and those with varying levels of balance and UL/LL impairments. Features like sensor straps and the ability to practice exercises from sitting or standing positions enabled broad participation. These characteristics of the Valedo system help overcome the limitations reported in previous literature that required participants to have a high balance ability or mild UL impairment to be able to participate in similar types of intervention [26,39]. However, the design of some games challenged the implementation of this intervention. Although the use of video games could limit the feeling of boredom resulting from conventional rehabilitation, games with monotonous repetitive movements, such as the Clock game, were perceived as “boring”, highlighting the need to customize game challenges to maintain engagement and motivation. These findings align with those of previous research, which identified the need for tailoring the level of challenge to match stroke participants’ abilities. They noted that participants’ disappointment and frustration were closely related to the challenges experienced (i.e., speed of reaction) during the playing of Wii video games [57]. For successful rehabilitation, personalizing an optimal task difficulty level according to individuals’ capacities has been suggested to ensure exercise engagement and thereby improve adherence [50,58]. However, the current study was unable to personalize each game for the participants because some factors, such as the speed of ball and randomness of fruits, could not be adjusted.

Even in the absence of cognitive deficits among study participants, cognitive challenges in some games (e.g., Break Breaker) influenced the feasibility of implementing this type of intervention. The high cognitive multitasking required by these games made them less preferred. The inability to master the required components of exercise could decrease exercise participation [59]. This suggests the necessity of considering cognitive abilities when designing video game interventions for stroke survivors. These findings were confirmed by a previous study, which revealed that poor cognitive ability impeded exercise engagement and was considered a barrier to using a game-based rehabilitation system in balance training post-stroke [50].

Multimodal feedback (audio and visual) provided by the Valedo system played a significant role in participant motivation. Feedback was perceived as informative and useful, with scores and rewards driving participants to improve their performance. However, some participants found certain feedback strategies unclear, suggesting the need for simple and understandable feedback. Existing literature suggests that extrinsic feedback (either audio or visual) in the form of knowledge of results and knowledge of performance are the key components in encouraging motivation [21,22]. Off-the-shelf commercial video game systems, such as Nintendo Wii, are advantageous for stroke rehabilitation because they are less expensive than robotic or immersive systems, are easy to set up, and provide performance feedback [26]. However, several studies have reported limitations with these systems, including insufficient, inaccurate, and disappointing feedback [26,60,61].

The current study found no major adverse events associated with Valedo video game-based trunk exercises. However, a minority of participants experienced fatigue and lateral trunk muscle tightness, which alleviated over time. Fatigue has been reported in other studies using video games for stroke rehabilitation [57,62,63]. The experience of muscle tightness could be due to reduced flexibility of trunk muscles, and this was reported in an RCT investigating the effect of core stability training among stroke patients. Future research should quantitatively measure fatigue and muscle flexibility to determine their clinical significance.

## 5. Limitations of the Study

While our results are promising, this study has several limitations that warrant cautious interpretation. The small sample size, participant heterogeneity, and absence of a control group limit the generalizability of our findings. Additionally, the games were in the development phase and could not be personalized to participants’ physical and cognitive abilities, making them challenging. For instance, the speed of the Break Breaker ball and the randomness of fruits in the Fruits game could not be adjusted, posing physical and cognitive challenges. Furthermore, the feedback provided by the games was not intuitive, requiring explanation and failing to guide participants on how to improve in future sessions.

## 6. Conclusions and Clinical Implications

The findings from this study demonstrate the feasibility of using Valedo video games to deliver trunk exercises for individuals with chronic stroke. However, several considerations, including physical and cognitive abilities, need to be taken into account to avoid inappropriate task difficulty and frustration. Only minimal resultant symptoms were reported throughout the intervention, including fatigue, temporary dizziness, and lateral trunk muscle tightness. Therefore, assessment of cognitive ability and muscle flexibility is recommended in future studies.

Clinicians might consider using this type of intervention to mitigate the boredom associated with conventional repetitive exercise. Further studies with larger sample size and control groups are warranted to compare the effects of video game-based trunk exercises with conventional trunk exercises. Additionally, technical adjustments to the video games are necessary to accommodate varying levels of physical and cognitive abilities.

## Figures and Tables

**Table 1:** Participant characteristics.

Site	ID	Age (Year)	Sex	Stroke Onset (Months)	Hemiplegic Side	TIS	BBS	AD
UK	P1	49	M	155	Left	14	52	None
	P2	54	F	75	Right	11	44	None
	P3	62	M	40	Left	12	24	W/C
	P4	69	M	169	Left	9	27	Cane
	P5	59	M	104	Left	16	46	None
KSA	P6	92	M	20	Right	11	25	Cane
	P7	75	M	21	Left	13	40	Cane
	P8	40	M	127	Left	11	45	None
	P9	75	M	53	Right	15	26	Cane
	P10	54	F	44	Right	12	11	W/C

UK = United Kingdom, KSA = Kingdom of Saudi Arabia; M = male; F= female; TIS = Trunk Impairment Scale, BBS = Berg Balance Scale; AD = assistive device; W/C = wheelchair.

**Table 2:** Psychosocial impact of PIADS scoring for each participant.

ID	P1	P2	P3	P4	P5	P6	P7	P8	P9	P10
Item	Score	Score	Score	Score	Score	Score	Score	Score	Score	Score
Competence	2	3	2	2	2	1	2	2	2	2
Happiness	3	3	0	3	2	3	3	3	2	3
Independence	2	3	0	3	2	1	1	2	0	3
Adequacy	1	3	0	2	2	1	3	0	2	3
Confusion	0	3	0	1	0	1	1	3	3	3
Efficiency	1	3	0	2	2	1	2	2	0	3
Self-esteem	2	3	0	1	2	3	3	3	0	3
Productivity	3	3	0	3	2	2	2	2	0	3
Security	2	3	0	3	0	2	2	2	2	3
Frustration	1	−3	0	2	0	0	−2	2	−2	0
Usefulness	1	2	1	2	2	0	1	2	0	0
Self-confidence	3	2	0	2	2	1	2	2	0	3
Expertise	2	2	1	2	2	0	2	0	0	0
Skilfulness	2	2	1	2	2	0	1	3	3	1
Well-being	3	3	0	2	2	3	3	3	0	3
Capability	1	2	1	2	2	1	2	3	0	0
Quality of life	2	3	0	2	1	2	1	0	0	2
Performance	3	2	0	3	1	2	2	2	2	2
Sense of power	3	3	0	3	2	2	2	3	1	3
Sense of control	3	3	0	3	1	0	2	0	2	3
Embarrassment	1	3	0	2	0	3	0	0	0	0
Willingness to take chances	1	3	0	1	2	2	2	3	2	3
Ability to participate	2	3	0	1	1	0	1	0	0	3
Eagerness to try new things	3	3	1	3	1	1	0	0	2	3
Ability to adapt to the activities of daily living	3	3	0	3	1	2	1	0	0	2
Ability to take advantage of opportunities	3	3	0	3	1	2	2	3	2	2
Total	51	54	7	52	37	28	39	39	17	50

**Table 3:** Participant adherence.

Site	ID	Planned Sessions	Attended Sessions	Completion Time	Percentage %
UK	P1	18	18	6 weeks	100
	P2	18	18	6 weeks	100
	P3	18	17	8 weeks	94.44
	P4	18	18	6 weeks	100
	P5	18	18	6 weeks	100
KSA	P6	18	18	6 weeks	100
	P7	18	18	8 weeks	100
	P8	18	18	8 weeks	100
	P9	18	12	8 weeks	66.67
	P10	18	18	6 weeks	100
Mean				6.8 weeks	96.11%

## Data Availability

The authors confirm that the data supporting the findings of this study are available within the article.

## Supplementary Materials

The following supporting information can be downloaded at: https://www.mdpi.com/article/10.3390/s24216830/s1, Table S1: Description of Valedo video games; Table S2: Identifying the level of a participant’s ability by researcher based on TIS and BBS results.
