# Supplementary material for: Video Game-Based Trunk Exercises for Rehabilitation in Chronic Stroke Survivors: A Mixed-Methods Feasibility Study
Source: Sensors (Basel). 2024 Oct 24;24(21):6830. doi: 10.3390/s24216830 (PMC11548624; doi:10.3390/s24216830)
Supplement: Supplementary file 1 [file sensors-24-06830-s001.zip › sensors-3217330-supplementary.pdf]

Supplementary material S1. Description of Valedo video games.

Game	Description
Diver<br>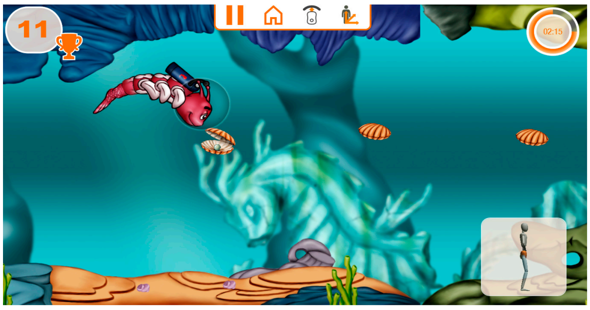	Goal of training: Trunk mobilization<br><br>Participant must execute fine, controlled trunk (flexion/ extension) or pelvic (anterior/posterior tilt) movements in a sagittal plane to make the caterpillar swim up and down to collects the shells and avoid the obstacles.
Glider<br>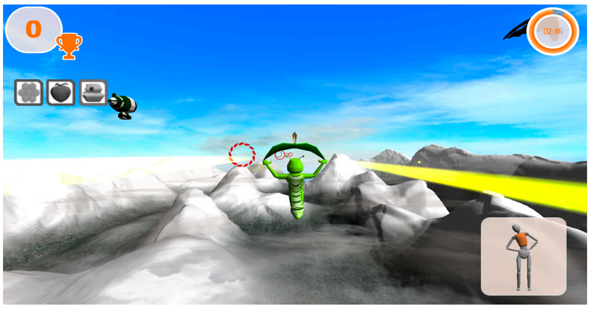	Goal of training: Trunk mobilization<br><br>Participant must execute fine, controlled combined movement of trunk (flexion/ extension and lateral flexion to left and right) or pelvic (anterior/posterior and left/right tilt) movements to fly up and down through the hoops.
Clock<br>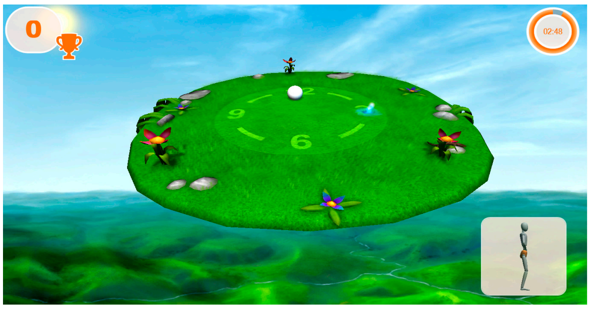	Goal of training: Movement awareness<br><br>Participant must execute fine trunk (trunk flexion/ extension) or pelvic (anterior/posterior tilt) movement to move the platform to the left and right, then return to the midline position.
Colours<br>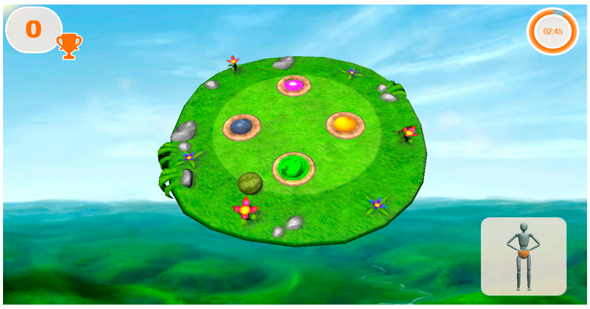	Goal of training: Trunk mobilization<br><br>Participant must execute fine combined movement of trunk (flexion/ extension and lateral flexion to left and right) or pelvic (anterior/posterior and left/right tilt) to move the ball toward the corresponding colour (e.g. green ball to green colour)

Brick-breaker<br>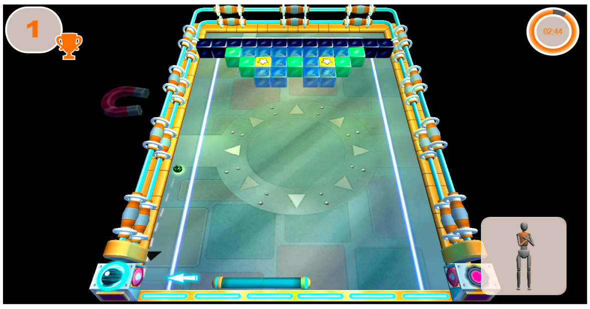 	Goal of training: Trunk mobilization<br><br>Participant must rotate trunk the left and right in a controlled manner to avoid losing the ball.
Fruits<br>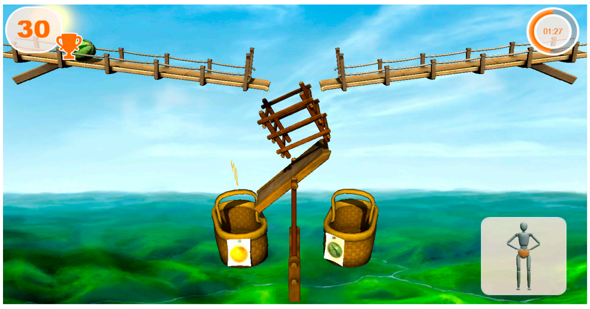 	Goal of training: Movement isolation<br><br>Participant must make controlled lateral pelvic tilt movements while keeping the trunk still to put every piece of fruit in a corrected respective basket (e.g. watermelon into the watermelon basket)

Supplementary material S2: Identifying the level of a participant's ability by researcher based on TIS and BBS results

Levels	Description	TIS and BBS scores	Games
Level 1	• Able to sit independently for over 10 seconds.<br>• Able to passively place and hold the non-paretic leg on the paretic leg.<br>• Able to actively place and hold the paretic leg on the non-paretic leg; backward displacement of the trunk over 10 cm is allowed.	Total TIS ≤ 6<br>BBS < 45	Diver<br>Glider<br>Clock<br>Brick-breaker<br><br>**Practice:** from sitting position
Level 2	• Able to sit independently for over 10 seconds.<br>• Able to passively place and hold the non-paretic leg on the paretic leg.<br>• Able to actively place and hold the paretic leg on the non-paretic leg; no backward displacement of the trunk over 10 cm.<br>• Able to touch bed/table with hemiplegic and non-hemiplegic elbow and return. Moves actively with or without compensations; appropriate shortening or lengthening is not necessary.	TIS static =7<br>TIS dynamic ≤ 6<br>BBS < 45	Diver<br>Glider<br>Clock<br>Colours<br>Brick-breaker<br>Fruits<br><br>**Practice:** from sitting position
Level 3	• Able to sit independently and appropriately shorten/lengthen to touch bed/table with hemiplegic and non-hemiplegic elbow.<br>• Shortening/lengthening when lifting the pelvis must not be correct.<br>• Asymmetrical rotation of the upper portion of the trunk.<br>• Static standing is safe.	TIS static =7<br>TIS dynamic ≤ 6<br>BBS ≥ 45	Diver<br>Glider<br>Clock<br>Colours<br>Brick-breaker<br>Fruits<br><br>**Practice:** from standing position
Level 4	• Able to lift the pelvis from bed/table without compensation.<br>• Rotation of the lower portion of the trunk is possible with or without compensation.<br>• Dynamic standing is safe.	TIS static =7<br>TIS dynamic =10<br>TIS coordination:1-2=4, 3-4 <3<br>BBS ≥ 45	Diver<br>Glider<br>Clock<br>Colours<br>Brick-breaker<br>Fruits<br><br>**Practice:** from standing position at medium to hard level
